# GABA-B receptors in the PNS have a role in Schwann cells differentiation?

**DOI:** 10.3389/fncel.2012.00068

**Published:** 2013-01-10

**Authors:** Patrizia Procacci, Marinella Ballabio, Luca F. Castelnovo, Cristina Mantovani, Valerio Magnaghi

**Affiliations:** ^1^Dipartimento di Scienze Biomediche per la Salute, Università degli Studi di MilanoMilan, Italy; ^2^Dipartimento di Scienze Farmacologiche e Biomolecolari, Università degli Studi di MilanoMilan, Italy

**Keywords:** GABA, actin, cAMP, myelin, non-myelinating cells

## Abstract

γ-aminobutyric acid type B (GABA-B) receptor mediates the inhibitory transmission of γ-aminobutyric acid in the mammalian nervous system, being present in neurons and also in glial cells. Recently the presence of GABA-B has been demonstrated in Schwann cells (SC) suggesting its contribution in regulating the cell fate, maturation, and plasticity. Here, we further support the functional presence of GABA-B receptor in SC plasma membrane. By confocal microscopy immunofluorescence we provide evidences that GABA-B localization on the cell elongated processes correlates with the morphological changes occurring in the differentiated SC. *In vivo* most of the GABA-B receptors seem to be present in non-myelinating SC, which are committed to ensheath the nociceptive fibers. Therefore, we argue that GABA-B receptors do not control exclusively the *in vivo* differentiation yielding the myelinating SC, but are also fundamental in regulating the SC plasticity *versus* the non-myelinating state. Data from the literature and our recent findings corroborate the role of the GABAergic system and GABA-B receptors in the peripheral nervous system, opening new perspectives on the mechanisms controlling the differentiation of SC.

## Introduction

Unlike neurons in the central nervous system (CNS), the peripheral nervous system (PNS) has the capacity to re-growth after a damage. This regenerative capacity mainly rests on the plasticity of Schwann cells (SC) and on their ability to switch between differentiation states (Jessen and Mirsky, 2005). SC, that normally produce an insulating myelin sheath around the axons, stimulate the survival and re-growth of injured nerves. Regenerative de- and re-differentiation as well as developmental differentiation, are tightly controlled by a balance of positive/negative regulators of SC maturation (Jessen and Mirsky, 2005, 2008). The comprehension of the mechanisms that contribute to SC plasticity are important not only to understand basic principles regulating SC biology and neuronal regeneration, but also because some of the molecules involved may act as modulators of peripheral neuropathies and associated neuropathic pain (Jessen and Mirsky, 2008). In recent years, the increased understanding of the cellular mechanisms controlling neuron-glial crosstalk in the PNS has focused on classic neurotransmitters, such as adenosine triphosphate (ATP) or γ-aminobutyric acid (GABA) (Fields and Stevens-Graham, 2002; Magnaghi et al., 2009). Here we survey the latest findings on the involvement of GABA system in SC, particularly via type B (GABA-B) receptor, assessing its potential as novel tool to regulate SC fate and plasticity.

## GABA-B receptor in the PNS

### GABA-B receptor

In the mammalian nervous system the GABA-B receptor is one of the native targets of GABA, the main inhibitory neurotransmitter (Bowery et al., 2002; Olsen and Sieghart, 2008). GABA-B is a distinct, baclofen-sensitive, metabotropic receptor, member of the seven transmembrane G-protein-coupled receptor superfamily. The intracellular signaling involves the activation of the adenylate cyclase second messenger systems and/or calcium and potassium channels activities (Bowery et al., 2002). GABA-B receptor is a heterodimer composed of two subunits, respectively GABA-B1 that is responsible for the ligand binding (Kaupmann et al., 1997, 1998; Jones et al., 1998; White et al., 1998; Kuner et al., 1999; Pfaff et al., 1999), and GABA-B2, which is necessary for the trafficking of the heterodimer to the cell surface and for signal transduction (Ng et al., 1999).

### GABA-B receptor in the PNS

The presence of GABA and its receptors in peripheral nerves date back to the 1980's (Brown and Marsh, 1978; Brown et al., 1979; Jessen et al., 1979; Morris et al., 1983; Olsen et al., 1984; Bhisitkul et al., 1987), but only recent findings yielded by reverse transcriptase-polymerase chain reaction, western blot, and immunohistochemistry, have clearly stated that GABA-B receptors are present in the PNS (Magnaghi et al., 2004, 2006; Magnaghi, 2007).

### GABA-B receptor in the PNS of knockout mice

The absence of GABA-B1 receptors has been confirmed in different nervous and non-nervous tissues of GABA-B1 knockout mice (Schuler et al., 2001). Hence, these mice are a good model to study the biochemical, morphological, and physiological consequences of the constitutive loss of GABA-B functions (Schuler et al., 2001). Recently, it has been shown that lack of GABA-B receptor activity in GABA-B1 knockout mice affects PNS myelination process, which in turn can contribute to the sensory phenotype observed (Magnaghi et al., 2008). Indeed, GABA-B1 deficient mice exhibited morphological and molecular changes in the peripheral myelin, including increases in the number of irregular fibers and in the expression levels of the myelin proteins, such as peripheral myelin protein 22 (PMP22) and myelin protein zero (P0). The density of small myelinated fibers and small neurons of the lumbar dorsal root ganglia (DRG) was raised in GABA-B1 deficient mice. These changes were also associated to altered gait behavior and pain perception, as well as with hyperalgesia to thermal stimuli (Magnaghi et al., 2008).

### GABA-B receptor in SC

In the PNS GABA-B receptors are mostly localized on the SC (Magnaghi et al., 2004; Magnaghi, 2007; Faroni et al., 2011). In these cells, GABA-B receptors proved to be negatively coupled to the adenylate cyclase system (Magnaghi et al., 2004), controlling SC proliferation and, overall, the myelin gene expression, both *in vitro* and *in vivo* (Magnaghi et al., 2004, 2008). Indeed, the activation of GABA-B receptor with the specific agonist baclofen counteracted the forskolin-induced SC proliferation at 5 days *in vitro*, with a reduction of the percentage of BrdUrd immunopositive SC (Magnaghi et al., 2004). In addition, GABA-B receptor activation decreased the expression levels of some myelin proteins, like PMP22, P0, connexin 32, and the myelin-associated glycoprotein (MAG) (Magnaghi et al., 2004). Given that the enzyme adenylate cyclase is able to regulate SC proliferation (Lee et al., 1999; Mirsky and Jessen, 1999) and the expression of specific myelin proteins (Suter et al., 1994; Scherer et al., 1995; Lee et al., 1999), it was conceivable to hypothesise that the GABA-B-induced decrease in cAMP levels accounts for the changes in cell proliferation and myelin gene expression in SC.

**Figure 1:**
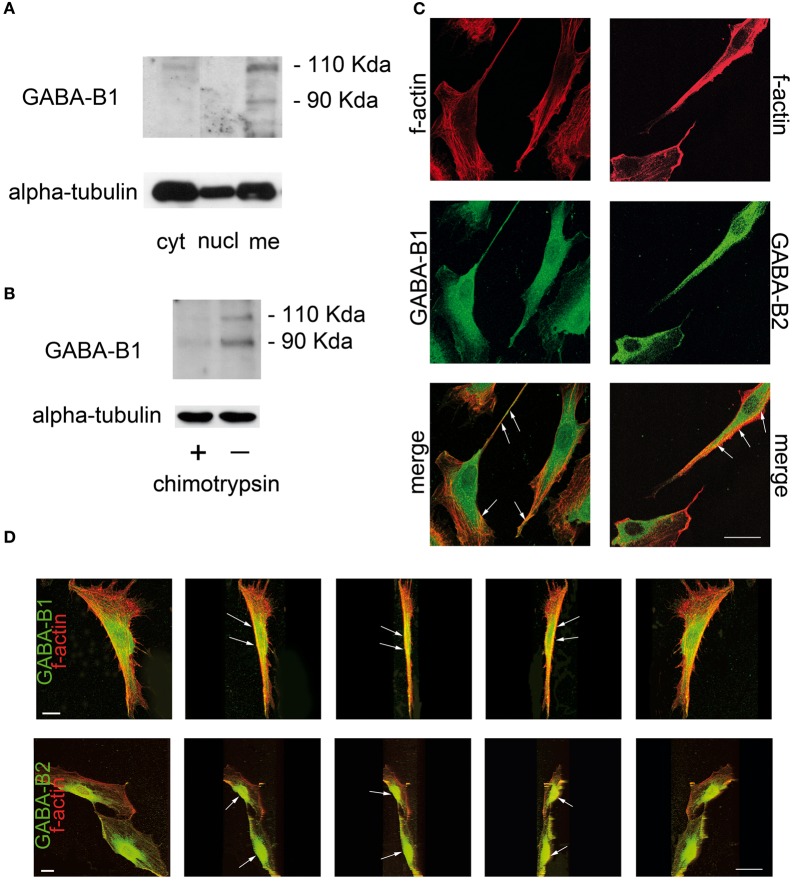
**GABA-B receptor is localized in plasma membranes of rat SC. (A)** SC cultures were grown in Dulbecco's modified Eagle's medium with 10% fetal calf serum and they were highly pure, as characterized with a specific protein marker S100. Western immunoblot was then performed on the different SC subcellular protein fractions (nuclear, cytosolic, and membrane-enriched) separated by ultracentrifugation at 100,000 g. The antibody anti-GABA-B1 [1:250 (Magnaghi et al., 2004)] recognized two bands of ~130 kDa and 100 kDa, corresponding to the 1a and 1b isoforms of native receptors in the membrane-enriched fraction of SC (Kaupmann et al., 1997; Ige et al., 2000). GABA-B1 was absent in the nuclear-enriched fraction, whereas two faint bands, likely corresponding to the GABA-B1 in the secretory granules, still appeared in the cytosolic-enriched fraction of SC. Equal loading was evaluated by western blot against alpha-tubulin. **(B)** GABA-B1 immunoblot of chymotrypsin-treated SC (+) showed very weak bands likely corresponding to the residual receptors in the cytoplasmic fraction, while two GABA-B1 bands were present in the whole protein extract from untreated SC (−). Equal protein loading was evaluated by immunoblot of alpha-tubulin. **(C)** Immunofluorescence and CLSM. The cellular cytoskeleton was revealed by phalloidin-TRITC staining of f-actin (1:250, in red). GABA-B receptor detection was done with Alexa-488 (in green). Merge images of GABA-B1 and GABA-B2 (in yellow) show that both receptor subunits are expressed throughout the SC and co-localize with f-actin at the cell border surface (arrows). **(D)** Progressive y-axis projections of SC merge images revealing that both GABA-B1 and GABA-B2 receptors were present at cell membrane surfaces (arrows) and ensheath the cell body compartments. Negative GABA-B controls for specificity showed only phalloidin-TRITC immunostaining (data not shown). Scale bars 20 μm.

However, the trafficking of the GABA-B receptor complex to the plasma membrane is also crucial for its functional activity (Bettler and Tiao, 2006). Recent studies evaluated the subcellular localization of GABA-B receptors in SC by protein fractioning and western blot analysis. Two bands of 130 kDa and 100 kDa, corresponding to the GABA-B 1a and 1b native isoforms respectively (Kaupmann et al., 1997; Ige et al., 2000), were found in the membrane-enriched fraction of SC. By contrast, very faint bands likely corresponding to the GABA-B1 in the secretory granules still persisted in the cytoplasmic fraction (Figure 1A). Accordingly, in SC treated with the enzyme chymotrypsin, which cleaves the peptidic bonds of proteins localized in the outer plasma membrane, the two bands corresponding to the GABA-B1 isoforms disappeared, whereas very weak bands still corresponding to the cytoplasmic fraction appeared (Figure 1B). A further evaluation of the GABA-B expression in SC membrane was done by immunofluorescence and confocal laser scanner microscopy (CLSM). f-actin is a protein producing a polymer that lies beneath the plasma membrane (Pollard and Borisy, 2003), and that is also present in lateral microvilli of SC (Trapp et al., 1989). As a valuable tool for f-actin labeling (Faulstich et al., 1983; Barber et al., 2004), the tetramethyl rhodamine isothiocyanate (TRITC)-fluorescent conjugate of phalloidin is useful to highlight the SC perimeter and shape (red in Figure 1C). GABA-B1 subunit was detected in SC cytoplasm and in cellular processes (green in Figure 1C), while GABA-B2 was found in SC body compartment (green in Figure 1C). Although GABA-B1 and GABA-B2 appeared generally spread throughout the SC body, the cytoplasmic presence was not unexpected because data from the literature indicates that GABA-B receptors may function as co-regulator of nuclear transcription factors such as ATF-4 or CREB-2 (Charles et al., 2003). However, merge images revealed that both GABA-B1 and GABA-B2 receptors were localized in the SC membranes, likely bounded to the f-actin filaments tracing the cells border (yellow in Figure 1C). By processing the y-axis projections of the confocal images of GABA-B/f-actin co-localizations, GABA-B1 and GABA-B2 resulted particularly enriched at the membrane surfaces ensheathing the cell body compartments (green in Figure 1D), although the GABA-B labeling was also present at the cell borders outlined by the f-actin cytoskeleton (yellow in Figure 1D). As expected, all these observations further confirmed the cell membrane localization of GABA-B receptors in SC.

### Does GABA-B have a role in SC differentiation?

SC committed in the process of myelination differentiate *in vivo* in response to contact with axons (Aguayo et al., 1976; Weinberg and Spencer, 1976). *In vitro* the same phenomenon of differentiation needs the presence of serum plus agents rising cAMP, like forskolin or 8Br-cAMP (Sobue et al., 1986; Morgan et al., 1991). In this context, agents modulating GABA-B receptors and in turn cAMP levels may thus participate in the control of SC differentiation and myelination processes.

The rearrangement of actin cytoskeleton is critical for SC differentiation and myelination (Fernandez-Valle et al., 1997; Gatto et al., 2003). During differentiation, SC change from a flat shape into a spindle-shaped phenotype; these cells present a reorganization in f-actin cytoskeleton and the appearance of bundles of stress fibers (Li et al., 2003). In such a way, the SC that assume the bipolar-shaped morphology resemble to the cells starting the myelination process *in vivo*.

**Figure 2:**
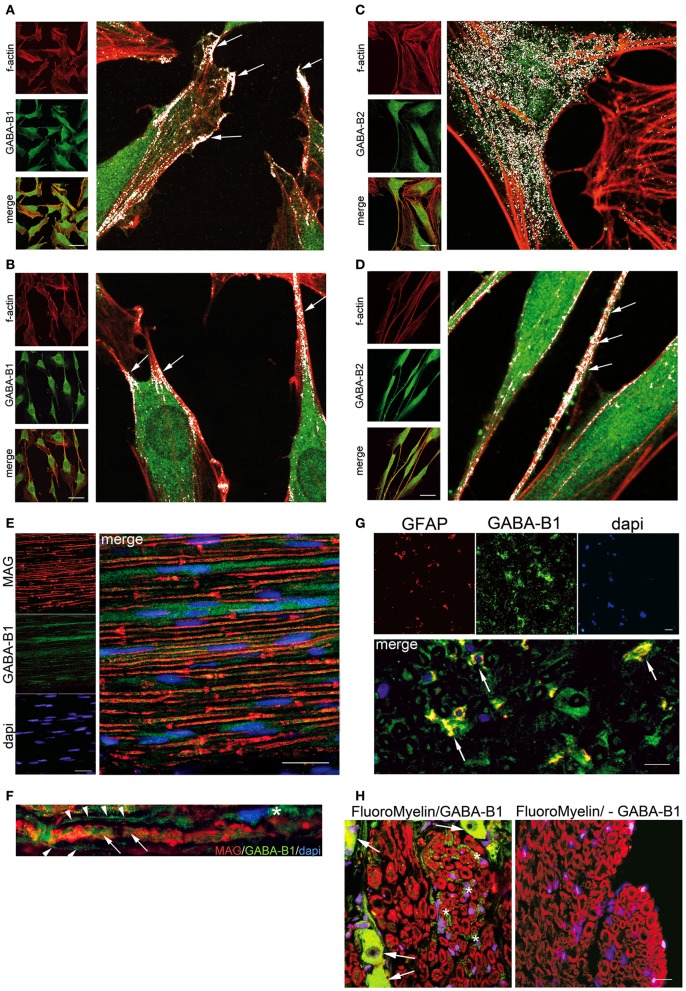
**GABA-B receptor localization changes in differentiated SC *in vitro* and *in vivo*.** Forskolin treatment (100 μM for 8 days)-induced strong f-actin cytoskeleton-based change in SC. Indeed, control SC (no forskolin) exhibited a polymerized f-actin wreath-like structure (**A,C** left panels in red), while forskolin treatment prompted the cells to present a stereotypical bipolar spindle-shape and elongated processes (**B,D** left panels in red). **(A)** and **(B)** Immunoflorescence for GABA-B1, **(C)** and **(D)** Immunoflorescence for GABA-B2, in undifferentiated and differentiated SC respectively. The GABA-B receptor and f-actin merge images revealed a different localization of GABA-B in the cell compartments, likely depending by high cAMP levels induced with forskolin. In all immunofluorecence the GABA-B detection was done with Alexa-488 (in green) while phalloidin-TRITC stained f-actin (1:250, in red). Merge images (in yellow) showing that f-actin/GABA-B co-localizations changed in distribution between undifferentiated and differentiated SC. Four high magnification images were done with LaserPix software (Bio-Rad Lab), based on Pearson's correlation coefficient with Costes'approach [reviewed in Bolte and Cordelieres (2006)]. LaserPix allows to superimpose a white mask onto the merge images, so that co-localized pixels of f-actin and GABA-B receptor are easily revealed as white dots. The average signal intensity/total pixels for white dots were calculated (104.15 undifferentiated vs. 92.24 differentiated SC, signal intensity/total pixels for GABA-B1; 106.91 undifferentiated vs. 83.54 differentiated SC, signal intensity/total pixels for GABA-B2). In undifferentiated SC GABA-B1 was present on cell protruding tips (arrows in **A** high magnification), while GABA-B2 was spread through the cytoplasm (**C** high magnification). After differentiation, the elongated processes of SC presented a white co-localization for both GABA-B receptors and f-actin (arrows in **B** and **D** high magnification). In these regions the co-localization of f-actin and GABA-B1 receptors was 94.1 ± 6.7%, while that of f-actin and GABA-B2 receptors was 86.3 ± 9.7%. GABA-B receptor was also analyzed in myelinating and non-myelinating SC. **(E)** Immunofluorescence *in vivo* in rat fibers of the sciatic nerve to evaluate the co-localization of GABA-B receptor and MAG [1:200, a specific protein of the adaxonal membrane of myelinating SC, (Scherer and Arroyo, 2002)]. Some GABA-B1 positive SC were distributed along axons (in green) without signs of co-localization with MAG (in red). **(F)** Same result was evident at high magnification, whereas GABA-B1 labeled the SC cytoplasm (asterisk), bordering the nerve fiber (arrowheads) as well as labeling the axon (arrows), but no sign of co-localization with MAG were shown. **(G)** In rat coronal section of rat sciatic nerve was assessed the co-localization of GABA-B receptor and GFAP [1:250, a specific marker of non-myelinating SC (Jessen and Mirsky, 1984)]. Several SC were double-stained for GABA-B1 (green) and GFAP (red) showing some co-localization in SC (yellow). **(H)** Immunofluorescence *in vivo* in rat saggital section of DRG to assess GABA-B receptor (in green) in the afferent sensitive fibers. Soma of sensitive neurons were GABA-B positive (arrows, left panel). The small sensitive fibers were labeled in red with FluoroMyelinTM (1:300). Several sensitive fibers were also immunopositive for GABA-B1 (green, left panel) with a preferential localization in non-myelinating SC (asterisk). No GABA-B1 was present in rat sciatic sections used as negative control (right panel, lack of GABA-B1 antibody). Nuclei were stained with dapi. Scale bars 20 μm.

Data collected in our laboratory, by means of co-localization studies (Bolte and Cordelieres, 2006), evidenced that the forskolin-induced SC differentiation correlates with a redistribution of GABA-B1 and GABA-B2 receptors on the SC surface. Indeed, in the undifferentiated SC (Figure 2A), the GABA-B1 receptor was localized on the protruding distal tips (arrows in Figure 2A magnification). When the SC differentiation starts, with changes in f-actin distribution and morphology (Figure 2B), GABA-B1 localized in the elongated regions, where the SC assumed the typical spindle-shaped morphology (arrows in Figure 2B magnification). In these selected regions the co-localization of f-actin and GABA-B1 receptors was 94.1 ± 6.7%. At the same time, in undifferentiated SC GABA-B2 showed a spread distribution (white in Figure 2C magnification), which turned to be localized in specific sites when, the SC differentiated and became elongated (Figure 2D). For instance, in these sites the f-actin boundaries were strictly assembled, SC cytoplasm extruded and the GABA-B2 receptor immunopositivity was very bright (arrows in Figure 2D magnification); the co-localization of f-actin and GABA-B2 receptors was 86.3 ± 9.7%.

The peculiar localization of GABA-B receptors on the elongations, tips, and bipolar processes, when the SC are induced to differentiate with forskolin, supports the proposed role of GABA-B receptors for SC differentiation. Moreover, this localization suggests “how” and “where” the GABA-B receptors are present. Both features may be an important requirement for the rearrangements associated to SC differentiation and for the establishment of the complex machinery regulating the myelination program *in vivo*.

The forskolin-induced GABA-B redistribution in SC seems to be specific, in contrast with the localization of neuregulins alpha and beta, which are differently affected by forskolin (Raabe et al., 2004). Indeed, in untreated SC both neuregulins are co-distributed throughout the cytoplasm, cell processes, and mostly excluded from the nucleus. After forskolin treatment the neuregulins change becoming either perinuclear or nuclear for alpha and beta, respectively (Raabe et al., 2004). In addition, the nerve growth factor (NGF) receptor is largely present at the distal ends of the SC, suggesting a supplementary function for this receptor during SC migration, development or ensheathing of axons (Assouline and Pantazis, 1989). However, the forskolin-induced GABA-B redistribution may be dependent from SC differentiation, but may be also a consequence of the reorganization of the SC actin cytoskeleton.

### GABA-B receptor is expressed in non-myelinating and/or myelinating SC

SC are very plastic cells that may switch between the undifferentiated and differentiated states. As a consequence SC may assume the non-myelinating and/or myelinating condition respectively (Jessen and Mirsky, 2005, 2008). This is relevant for the axon-glial crosstalk, for the development of sensory or motor functions, and for nerve regeneration after an injury.

The findings gathered from GABA-B1 knockout mice suggest the importance of GABA-B in non-myelinating SC regulating the sensory functions (Magnaghi et al., 2008), nevertheless the literature in this regard is still poor.

In theory, the SC that myelinate *in vivo* are generally considered comparable to SC that differentiate *in vitro*. In this context, rat nerve fibers were evaluated for the co-localization of GABA-B1 receptor and MAG, a specific protein localized on the inner/adaxonal membrane in the myelinating SC and in the Schmidt-Lanterman incisures of non-compact myelin (Scherer and Arroyo, 2002). Some GABA-B1 positive SC were present in the nerve, distributed along axons (Figure 2E in green) but no significant co-localization with MAG (in red) was evident, neither in the adaxonal membrane nor in the Schmidt-Lanterman incisures (Figures 2E,F in yellow). The lack of co-localization of GABA-B and MAG *in vivo*, indicates that GABA-B receptors are not preferentially involved in the SC committed to myelinate. Indeed, several SC in the coronal section of rat sciatic nerve were double-stained for GABA-B1 (green) and for the glial fibrillary acidic protein (GFAP, in red) showing co-localizations in SC (yellow in Figure 2G). Given that GFAP is a marker of non-myelinating SC in the PNS (Jessen and Mirsky, 1984), this result strength the preferential localization of GABA-B1 in these SC deputed to surround the axons of small sensitive neurons.

The localization of GABA-B1 in non-myelinating SC was further supported by the analysis of sensory DRG. The non-myelinated fibers are generally known to be sensitive fibers entering the DRG (Markenson, 1996). As expected, analyzing the coronal sections just at the border between the sensitive dorsal root and the DRG structure, the soma of some sensory neurons of DRG showed a bright immunopositivity for GABA-B receptors (arrows in Figure 2H, left panel). Contemporarily, also several sensitive fibers entering the DRG, which are light myelinated fibers (A delta fibers) or non-myelinated fibers (C fibers), were GABA-B1-immunopositive (green Figure 2H, left panel). However, the GABA-B1-immunopositive fibers were not tightly connected to the myelin rings (red Figure 2H, left panel) corroborating the preferential GABA-B1 localization in the non-myelinating SC that surround the groups of axons (green Figure 2H, left panel).

Although other analysis are needed to support this hypothesis, our results indicate an interesting participation of GABA-B receptors in the differentiation/de-differentiation processes occurring in SC. Indeed, the GABA-B receptors at the SC tips and edges may be relevant when the SC are committed to become non-myelinating SC.

## Conclusion

The observations presented in this paper, gathered from the literature and from our findings, stated that GABA-B receptors are present and functional active in SC of the PNS. The GABA-B receptor implication in the commitment of SC into the myelination program might represent a fascinating functional significance for their localization on the elongated parts of the differentiated SC. However, the GABA-B-mediated control of the differentiation/de-differentiation may be primarily relevant when SC are committed to become non-myelinating SC, thus forming the peripheral nociceptive fibers. In our opinion this is the main significance of GABA-B1 presence in SC. The efforts are now toward the perspective to demonstrate the role of GABA-B1 in non-myelinating SC. However, some questions are still open. Does a specific GABA-B1 receptor ablation affect SC or sensory neurons? Is the GABA-B receptor expression developmentally regulated? Does it co-localize with other different GABA receptors and/or proteins? The answers to these questions will shed light on the complexity of the functional presence of GABA-B receptors in the PNS.

### Conflict of interest statement

The authors declare that the research was conducted in the absence of any commercial or financial relationships that could be construed as a potential conflict of interest.
